# *Leishmania* (*Leishmania*) *mexicana* Infection in Wild Rodents from an Emergent Focus of Cutaneous Leishmaniasis in Yucatan, Mexico

**DOI:** 10.1155/2022/8392005

**Published:** 2022-05-31

**Authors:** Erika I. Sosa-Bibiano, Luis A. Sánchez -Martínez, Karina B. López-Ávila, Juan B. Chablé-Santos, Jimmy R. Torres-Castro, Edith A. Fernández-Figueroa, Claudia Rangel-Escareño, Elsy N. Loría-Cervera

**Affiliations:** ^1^Laboratorio de Inmunología, Centro de Investigaciones Regionales “Dr. Hideyo Noguchi”, Universidad Autónoma de Yucatán, Mérida, Yucatán, Mexico; ^2^Departamento de Zoología, Facultad de Medicina Veterinaria y Zootecnia, Campus Ciencias Biológicas y Agropecuarias, Universidad Autónoma de Yucatán, Mérida, Yucatán, Mexico; ^3^Dirección de Prevención y Protección de la Salud de los Servicios de Salud del Estado de Yucatán, Mérida, Mexico; ^4^Computational Genomics and Integrative Biology, National Institute of Genomic Medicine, Periférico Sur 4809 Arenal Tepepan, Ciudad de México 14610, Mexico; ^5^School of Engineering and Sciences, Tecnológico de Monterrey, Epigmenio González 500, San Pablo, Santiago de Querétaro 76130, Qro, Mexico

## Abstract

In 2015, emergent cases of localized cutaneous leishmaniasis (LCL) were reported in Tinum, Yucatan, Mexico. As part of an eco-epidemiological study to characterize the elements that trigger *Leishmania* infection in that area, we conducted a field study to investigate the occurrence of *Leishmania* infection in wild rodents. From November 2019 to February 2020, rodents were caught from three sites located in the municipality of Tinum, Yucatan. For each specimen, clinical signs suggestive of *Leishmania* infection were recorded. Samples from the tail, liver, and spleen were taken for the identification of *Leishmania* DNA by PCR. Twenty rodents belonging to two species were caught including *Heteromys gaumeri* (55%, 11/20) and *Ototylomys phyllotis* (45%, 9/20). Fifty-five percent of the animals presented white spots on the tail, 15% had splenomegaly, and 5% had hepatomegaly. Fifty-five percent (11/20) of the animals were found infected by *Leishmania*. *Heteromys gaumeri* was caught in all trapping sites and was the most infected species (63.6%, 7/11). The percentage of infection for *O*. *phyllotis* was 44.4% (4/9). *Leishmania* (*Leishmania*) *mexicana* was identified as the infecting species in two *H*. *gaumeri*. This study provides, for the first time, evidence of *Leishmania* infection in wild rodents from the Yucatan state. *Heteromys gaumeri* and *O*. *phyllotis* may be involved in the transmission cycle of *L*. *mexicana* in this emergent focus; however, further longitudinal studies are needed to confirm their role as primary reservoirs.

## 1. Introduction

Parasites from the genus *Leishmania* (Kinetoplastea: Trypanosomatida) are the causal agents of the leishmaniases, a group of tropical diseases transmitted by the bite of Phlebotomine sand flies (Diptera: Psychodidae). Approximately, 53 *Leishmania* species have been described from different regions of the world; of these, 31 species are known to be parasites of mammals and 20 species are pathogenic for human beings [1]. The disease is manifested by three major clinical forms: localized cutaneous leishmaniasis (LCL), mucocutaneous leishmaniasis (MCL), and visceral leishmaniasis (VL). Today, more than 1 billion people in 98 countries live in endemic areas and are at risk of infection. In addition, an estimated 0.2–0.4 million VL cases and 0.7–1.2 million CL cases occur each year [2, 3].

Leishmaniases are zoonotic diseases where mammals play a crucial role in the transmission cycle [4]. Many natural hosts of *Leishmania* parasites have been identified including foxes, coatis, raccoons, opossums, bats, monkeys, dogs, and rodents [4, 5]. Some of them have been incriminated as potential reservoirs due to their abundance, life expectancy, temporal, and spatial co-occurrence with vectors and their long co-evolution with *Leishmania* species [6]. The identification of *Leishmania* carriers and the study of their role as potential reservoirs are very important to understanding the epidemiology of the disease, especially in areas where leishmaniasis is emerging [7].

Localized cutaneous leishmaniasis (LCL) is endemic in Mexico, mainly in the states of Veracruz, Tabasco, Oaxaca, Chiapas, Campeche, and Quintana Roo [8]. The disease could be asymptomatic or manifest as an ulcer with raised borders located mainly in the ear (39.8%) [8–10]. Most studies to characterize the eco-epidemiological features of LCL have been conducted in the state of Campeche [10]. According to those studies, the main causal agent is *Leishmania* (*Leishmania*) *mexicana* and the only proven vector is *Lutzomyia olmeca olmeca*. However, *Lu*. *cruciata*, *Lu*. *panamensi*, and *Lu*. *shannoni* have been incriminated as potential vectors of *L*. (*L*.) *mexicana* [11–13]. In Campeche, *Heteromys gaumeri*, *Peromyscus yucatanicus*, and *Ototylomys phyllotis* were incriminated as *L*. (*L*.) *mexicana* reservoirs based on their geographical and temporal distributions and their overlap with vector and human habitats [14–16].

In the Yucatan State, cases of LCL had been restricted to sylvatic areas of the southern cone, close to the well-known endemic areas of Campeche and Quintana Roo [17, 18]. Nevertheless, since 2015, the Health Services of Yucatan have reported emergent cases in rural communities of the municipality of Tinum, Yucatan, located in the eastern part of the state [19]. This emergency has prompted us to carry out an eco-epidemiological study to identify all the transmission components (*Leishmania*, vectors, and reservoir species) in this area. As a first step to identifying potential reservoirs in Yucatan, we investigate the occurrence of *Leishmania* infection in wild rodents from two villages with autochthonous cases of LCL in the municipality of Tinum, Yucatan, Mexico.

## 2. Materials and Methods

### 2.1. Study Area

**Figure 1:**
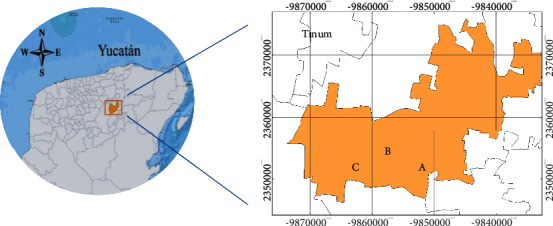
Geographical locations of the trapping sites in the municipality of Tinum, Yucatan. (a) A forest fragment. (b) An abandoned cornfield. (c) A perirural site.

Rodents were caught in two villages of the municipality of Tinum, Yucatan; located between 20°40′ and 20°53′ latitudes North and 88°21′ and 88°33′ longitudes West. Trapping sites (Figure 1) were selected according to previous reports of human *Leishmania* infection in the municipality of Tinum [19] including a forest fragment located at 20.66°N, 88.49°W; an abandoned cornfield located at 20.67°N, 88.47°W; and a perirural site located at 20.64°N, 88.51°W. The area has an annual rainfall that reaches 85.9 mm mostly during the summer, an average temperature of 26.3°C, and vegetation corresponding to the dry medium-height semideciduous forest with a predominance of cedar (*Cedrus odorata*), mahogany (*Swietenia macrophylla*), ceiba (*Ceiba pentandra*), chacá (*Bursera simaruba*), and pochote (*Bombacopsis quinata*) [20].

### 2.2. Collection of Rodents and Tissue Sampling

Trapping was carried out in November and December 2019 and February 2020 (a period where a strong correlation was demonstrated between the abundance and rates of infection of both reservoirs and vectors in endemic areas from Southeastern Mexico) [21]. Small rodents were caught alive in Sherman traps baited with corn and sunflower seeds. Ninety traps were laid at 10 m intervals during seven nights in the perirural site (where most clinical and asymptomatic human *Leishmania* infections were recorded), during four nights in the abandoned cornfield, and during three nights in the forest fragment. Traps were set in the evening in common rodent microhabitats such as burrows hidden in rocks and buttresses of trees and checked the following morning. The captures were conducted with the permission of the Mexican Secretariat of Environment and Natural Resources (SEMARNAT, license no. 12783) and approved by the Ethics Research Committee of the Autonomous University of Yucatan with the ID: CEI-22-2018.

Each rodent was identified into species according to its external characteristics using the keys of Hall [22] and Jones [23]. Then, they were examined searching for clinical signs suggestive of *Leishmania* infection such as ulcers, scars, white spots, and/or swelling of the tail [14]. Animals were euthanized by intraperitoneal injection of sodium pentobarbital (100 mg/Kg). Needle aspirates were taken from the base of the tail of each rodent, since it has been reported to be the site where ulcers commonly appear [14, 16]. Aspirates were cultivated in RPMI 1640 medium and incubated at 23.5°C. A culture was considered positive when at least one promastigote was observed microscopically and negative if no parasites were found within one month. Additionally, skin, spleen, and liver fragments were collected and frozen to −70°C until used.

### 2.3. DNA Extraction and Detection of *Leishmania* Infection

DNA was extracted from the different tissues using the standard phenol/chloroform method. The PCR reactions were performed with the GoTaq® PCR Core Systems I kit (Promega M7660) in a final volume of 25 *μ*L containing buffer 1x, 1.75 mM MgCl_2_, 0.2 mM dNTP's, 2.5 U/*μ*L Taq polymerase, 1 *μ*M of each primer JW11 (5′-CCTATTTTACACCAACCCCCAGT-3′), and JW12 (5′-GGGTAGGGGCGTTCTGCGAAA-3′), which amplify a 120 bp fragment of kDNA of the genus *Leishmania* [24] and 200 ng of DNA. The DNA isolated either from the footpad of an infected hamster or from parasites in culture was used as a positive control. As a negative control, DNA from the skin tail of a non-infected *Peromyscus yucatanicus* was used. Negative controls that contained water instead of DNA were also included in each PCR experiment. PCR amplification was performed in a SimpliAmp thermal cycler (Applied Biosystems) with an initial denaturation step at 94°C for 1 min to activate the Hot Start Taq polymerase followed by 30 cycles of 94°C for 1 min, 67°C for 30 s, and 72°C for 30 s with a final extension at 72°C for 5 min. The reaction products were run in 2.5% agarose gel stained with ethidium bromide (0.5 *μ*g/*μ*L) and visualized under UV light.

Sample typification to determine *Leishmania* species was carried out by PCR-RFLP. For the PCR analysis, a 300–350 bp of the internal transcribed spacer 1 (ITS1) was amplified using 10 *μ*L QIAGEN Master mix (QIAGEN Inc., Hilden, Germany), 1 *μ*L (10 *μ*M) of each primer ITS1 (F: 5′-CTGGATCATTTTCCGATG-3′ and R: 5′-TGATACCACTTATCGCACTT-3′), and 5 *μ*L of extracted DNA and RNAse free water in a final volume of 20 *μ*L. Additionally, *L*. (*L*.) *mexicana* (MHOM/MX/2011/Lacandona), *L*. (*L*.) *amazonensis* (MHOM/BR/1973/M2269), *L*. (*V*.) *braziliensis* (MHOM/BR/1995/M15280), and *L*. (*L*.) *infantum chagasi* (MHOM/BR/72/BH46) were used as positive controls. RNAse free water was used as a negative control. The PCR assay used the following amplification cycle: 94°C for 4 min followed by 36 cycles at 94°C for 40 s, 54°C for 30 s, and 72°C for 1 min and a final extension at 72°C for 6 min. Then, 3 *μ*L of the PCR product was reamplified following the amplification cycle: 94°C for 4 min followed by 18 cycles at 94°C for 40 s, 54°C for 30 s, and 72°C for 1 min and a final extension at 72°C for 6 min. The PCR products were run on a 1.5% agarose gel stained with SYBR Gold Nucleic Acid Gel Stain (S11494, Invitrogen, USA).

The PCR products from the reamplification of ITS were subjected to digestion with HaeIII restriction enzyme (R0108, New England, BioLabs) for *Leishmania* species identification. A mixture with 2 *μ*L of RNAse free water, 2 *μ*L of buffer, and 1 *μ*L of HaeIII was added to 15 *μ*l of the amplified product. Then, the microtube was incubated at 37°C for 3 hours and at 80°C for 20 min to inactivate the enzyme. The species profiles of each sample and reference controls were observed in a 4% agarose gel subjected to electrophoresis for 3.5 hours at 90 V. The gel was stained with SYBR Gold Nucleic Acid Gel Stain (S11494, Invitrogen, USA).

## 3. Results and Discussion

According to the WHO, surveys of mammals in new foci of *Leishmania* infection are essential to identify potential reservoir hosts and to understand the eco-epidemiology of the disease [6]. In this work, we reported, for the first time, *L*. (*L*.) *mexicana* infection in wild rodents captured in a rural community of the Yucatan state where autochthonous LCL cases emerged in 2015 [19].

**Figure 2:**
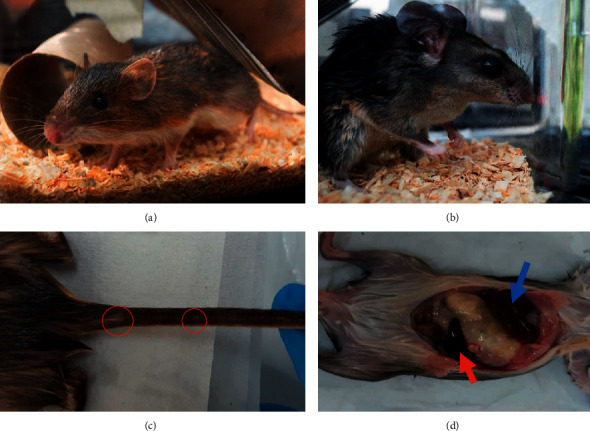
Illustrations of the *Heteromys gaumeri* (a) and *Ototylomys phyllotis* (b) specimens caught in Tinum, Yucatan. White spots suggestive of *Leishmania* infection in the tail of one *H*. *gaumeri* (c) and hepatomegaly (blue arrow) and splenomegaly (red arrow) in one *O*. *phyllotis* (d).

A total of twenty animals belonging to both the Heteromyidae and Cricetidae families were captured. The sample was represented by two rodent species (Figures 2(a) and 2(b)): eleven *Heteromys gaumeri* (55%) and nine *Ototylomys phyllotis* (45%). This finding is quite different from previous studies carried out in the sylvatic region of Campeche [14, 16], where a great diversity of small mammals has been reported, including *Oryzomys melanotis*, *Or*. *couesi*, *Sigmodon hispidus*, *Reithrodontomys gracilis*, *H*. *gaumeri*, *O*. *phyllotis*, *P*. *yucatanicus*, and the marsupial *Marmosa mexicana*. This result could be related to some ecological aspects of the state of Yucatan that are different from the entities that constitute the Yucatan peninsula, including its drier climate, the predominance of arid vegetation, and the high percentage of deforested areas [25–27]. However, limitations due to the catch effort cannot be excluded.

In this study, the same number of males and females were caught, and at the time of inspection, no animal had ulcers suggestive of *Leishmania* infection. However, 55% (11/20) of the animals presented white spots on the tail, 15% had splenomegaly, and 5% had hepatomegaly (Figures 2(c) and 2(d)).

**Figure 3:**
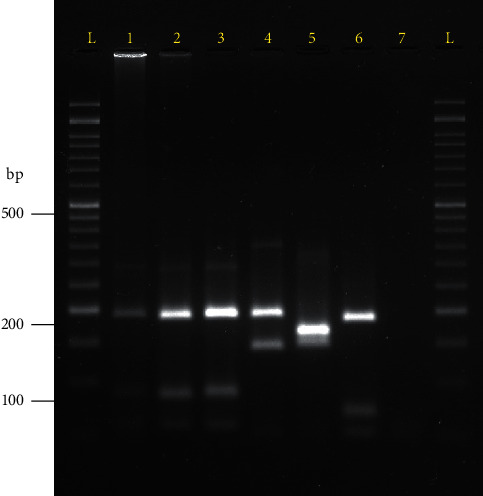
Restriction fragment length polymorphism (RLFP) patterns obtained from liver samples of two *H*. *gaumeri* (Hg). L, 100 bp molecular weight ladder; 1, Hg1 positive to *L*. *mexicana*; 2, Hg2 positive to *L*. *mexicana*; 3, reference strain of *L*. (*L*.) *mexicana* (MHOM/MX/2011/Lacandona); 4, reference strain of *L*. (*L*.) *amazonensis* (MHOM/BR/1973/M2269); 5, reference strain of *L*. (*V*.) *braziliensis* (MHOM/BR/1995/M15280); 6, reference strain of *L. (L.) infantum chagasi* (MHOM/BR/72/BH46); 7, negative control.

Parasites were successfully isolated from the base of the tail of one *O*. *phyllotis*. *Leishmania* DNA was detected in 55% (11/20) of the animals including seven *H*. *gaumeri* (out of 11 captured, 63.6%) and four *O*. *phyllotis* (out of 9 captured, 44.4%). Regarding the presence of parasite DNA in the different organs (Table 1), one *O*. *phyllotis* was positive in the skin, the liver, and the spleen; two *H*. *gaumeri* and one *O*. *phyllotis* were positive in the liver and the spleen; and three *H*. *gaumeri* and one *O*. *phyllotis* were positive only in the spleen. Only 27.27% (3/11) of the animals were positive in the skin (Table 1). *Leishmania* (*L*.) *mexicana* was detected as the infecting species in the liver samples from two *H*. *gaumeri* (Figure 3).

*Ototylomys phyllotis* and *H*. *gaumeri* are the two most abundant rodent species captured in the sylvatic endemic areas of LCL in Campeche [14, 16]. Moreover, several parasitological and mastozoological studies have incriminated these two species as potential reservoirs of *L. (L.) mexicana* in that area [11, 14, 16].

On the other hand, *O*. *phyllotis* was one of the first *Leishmania* carriers identified in sylvatic areas of the Yucatan Peninsula presenting multiple, hairless, white spots on the proximal half of the tail [14]. Later, its ability to retain *Leishmania* parasites for at least 18 months was demonstrated, confirming their role as a reservoir [15]. Tail depigmentation in *H*. *gaumeri* specimens was also frequently reported in surveys conducted between 1997 and 2004 in Calakmul, Campeche [16]. In this study, we found white spots on the tail in 55% (11/20) of the animals, and we detected *Leishmania* infection by PCR in 54.5% (6/11) of them, confirming that this sign could be considered as a fair indicator of *Leishmania* infection in wild rodents. Surprisingly, only three animals were positive to *Leishmania* DNA in the skin, the site where signs commonly appear.

The dissemination of several dermotropic *Leishmania* parasites to visceral organs in rodents has been reported previously. Tonelli et al. [28] found the DNA of *L*. (*V*.) *braziliensis* in the liver and the spleen of rodents captured in an ecotourism area from Brazil. Similarly, the presence of *L*. (*L*.) *tropica* (a species associated mainly with cutaneous manifestations in humans) was detected by PCR in the spleen of wild rodents captured in Ethiopia [29]. The systemic dissemination of *L*. (*L*.) *mexicana* in naturally infected rodents from one of the main LCL foci in Campeche, Mexico, was also documented [15]. Nevertheless, the presence of *L*. (*L*.) *mexicana* amastigotes in the organs (liver, spleen, kidney, and heart) of the rodents was not considered as a visceralization of the disease since its effect was nonpathogenic. In addition, the experimental infection of *Thrichomys laurentus*, a putative reservoir of *L*. (*V*.) *braziliensis* and *L*. (*L*.) *infantum* in Brazil, did not reveal histological changes in organ's tissues despite the presence of *Leishmania* DNA, suggesting that the division between cutaneous and visceral tropisms observed in humans is less clear in natural hosts of *Leishmania* spp. [30].

The association of parasite DNA in the liver and the spleen of rodents captured in the emergent focus of Yucatan with its pathogenic effects in the tissue needs to be investigated. Further studies are needed to understand this phenomenon and its implications in the natural transmission of *Leishmania* in endemic areas.

Regarding the infection rate by rodent species, here, we report that *H*. *gaumeri* is the species most infected by *Leishmania* (63.6%, 7/11). This finding is consistent with previous studies done in Campeche, where high infection rates (29–88%) have been documented for this Yucatan Peninsula's endemic species [16]. Due to their high abundances and infection rates, *H*. *gaumeri* has been incriminated as one of the three potential reservoirs of *L*. (*L*.) *mexicana* in Campeche [16]. On the other hand, several studies have provided evidence to incriminate *O*. *phyllotis* as a potential reservoir of *L*. (*L*.) *mexicana* in Mexico and Belize. In the latter, a prevalence of *Leishmania* infection ranging from 2% to 40% was reported for this species of animals [31–33]. In addition, the high attraction of *O*. *phyllotis* to *Lu*. *flaviscutellata* (the most important vector in Belize) has been proved, indicating its role as the main reservoir of *L*. (*L*.) *mexicana* in that country [34]. In Campeche, the *Leishmania* infection rate for *O*. *phyllotis* varied from 27% to 100%. *Peromyscus yucatanicus*, the other wild rodent incriminated as a reservoir of *L*. (*L*.) *mexicana* in Campeche, appear not to be involved in the transmission of the parasite in this new emergent focus of leishmaniasis at the Yucatan State [15, 16].

Here, we found *H*. *gaumeri* specimens in all trapping sites being more frequent (54.5%, 6/11) in the forest fragment followed by the perirural site (36.3%, 4/11). The peak of the abundance of this species was documented in November. *Ototylomys phyllotis* specimens were captured with a similar frequency in the abandoned cornfield (55.5%, 5/9) and the perirural site (44.4%, 4/9). The peak of the abundance of this species was documented in February.

Most of the infected animals (54.5%, 6/11) were found in the forest fragment followed by the abandoned cornfield (27.2%, 3/11) and the perirural site (18.2%, 2/11) (Table 2). *Heteromys gaumeri* was the most infected species (100%, 6/6) captured in the forest fragment suggesting that cutaneous leishmaniasis remains mainly a sylvatic zoonotic disease in the Yucatan State. The high infection rate for *O*. *phyllotis* (60%, 3/5) was observed in the abandoned cornfield (Table 2), and the similar proportions of the animals caught in both the abandoned cornfield and the perirural site suggest that this species could be maintaining the *L*. (*L*.) *mexicana* transmission cycle in human-disturbed areas.

## 4. Conclusion

Our results suggest that *H*. *gaumeri* and *O*. *phyllotis* are maintaining the *L*. (*L*.) *mexicana* transmission cycle in Yucatan, Mexico. However, further longitudinal studies are needed to confirm both their participation in LCL emergence and their role as reservoirs of *L*. (*L*.) *mexicana* in Tinum, Yucatan. According to our results, *H*. *gaumeri* could be involved in the sylvatic transmission cycle, while *O*. *phyllotis* could be maintaining the transmission cycle in disturbed areas. These findings are relevant for the development of prevention strategies in the municipality of Tinum, Yucatan.

## Figures and Tables

**Table 1:** Molecular results for *Leishmania* infection in the different tissues.

Animal ID	Species	PCR result by tissue		
		Skin	Liver	Spleen
01	Op	−	−	−
02	Hg	−	+	+
03	Hg	−	−	+
04	Hg	−	−	+
05	Hg	−	−	+
06	Hg	−	+	−
07	Hg	+	−	−
08	Op	−	−	−
09	Op	−	−	+
10	Op	−	+	+
11	Hg	−	−	−
12	Hg	−	−	−
13	Hg	−	−	−
14	Op	−	−	−
15	Op	+	+	+
16	Op	−	−	−
17	Op	+	−	+
18	Hg	−	−	−
19	Op	−	−	−
20	Hg	−	+	+

Hg, *Heteromys gaumeri*; Op, *Ototylomys phyllotis*; −, negative sample; +, positive sample.

**Table 2:** Distribution of rodent species with *Leishmania* infection by trapping site.

Species	Forest	PCR (+)	Cornfield	PCR (+)	Perirural site	PCR (+)
*Heteromysgaumeri*	6	6	1	0	4	1
*Ototylomysphyllotis*	0	0	5	3	4	1

## Data Availability

The data used to support the findings of this study are available from the corresponding author upon request.
